# Dietary Antioxidants and Chronic Diseases

**DOI:** 10.3390/antiox12020362

**Published:** 2023-02-02

**Authors:** Małgorzata Elżbieta Zujko, Anna Maria Witkowska

**Affiliations:** Department of Food Biotechnology, Faculty of Health Sciences, Medical University of Bialystok, Szpitalna 37, 15-295 Bialystok, Poland

Chronic diseases, most notably diabetes, cancer, cardiovascular diseases, neurodegenerative diseases, thyroid diseases, and allergic diseases are major causes of death, disability, and a lower quality of life in various populations.

Oxidative stress, defined as an imbalance between the production of pro-oxidants (reactive oxygen, nitrogen, and chlorine species) and the body’s production of antioxidants (enzymatic, e.g., superoxide dismutase, glutathione peroxidase, catalase, and non-enzymatic, e.g., glutathione, uric acid, melatonin, metal-binding proteins, bilirubin, polyamines), plays an important role in the pathogenesis of these diseases.

Lifestyle modifications, including a healthy diet, are a major therapeutic strategy in preventing and treating chronic diseases. Dietary antioxidants such as polyphenols, e.g., flavonoids (flavones, flavonols, theaflavins, catechins, proanthocyanidins, flavanones, anthocyanidins, and isoflavones) and non-flavonoids (phenolic acids, stilbenes, and lignans), antioxidant vitamins (C, E, A, carotenoids) and minerals (Se, Mn, Zn, Cu, Fe) can support the action of endogenous antioxidants in alleviating the negative effects of oxidative stress [1].

A new approach to a healthy diet is to asses dietary total antioxidant capacity (DTAC), because the whole diet contains various antioxidants with additive or synergistic effects. Several assays are available to measure antioxidants in foods, but the largest database is based on the FRAP (ferric ion-reducing antioxidant potential) method. Dietary FRAP has been shown to positively correlate with well-known indicators of a healthy diet [2]. Moreover, habitual antioxidant intake is significantly positively associated with the lifestyle and socioeconomic status of the population [3]. However, individual diet modification in terms of higher antioxidant intake can improve the clinical parameters of patients with metabolic syndrome (MetS), even before pharmacological treatment [4].

Paulis and Giorgio [5], in a report on three cases, demonstrated the positive effect of combined therapy involving oral intake of antioxidants and pharmacological treatment in urological diseases. Similarly, Candellone et al. [6] indicated that antioxidant supplementation (vitamin E, curcumin, resveratrol, quercetin) has a synergistic effect on the course of pharmacological treatment of thyroid diseases in both animals and humans.

In the Bialystok PLUS population in Poland, a higher quartile of DTAC, after adjustment for confounding variables, was significantly associated with a reduced odds ratio for the prevalence of prediabetes, and was inversely associated with HOMA-IR in a multivariate linear regression model. Moreover, DTAC was positively related to individual dietary antioxidants (polyphenols, antioxidant vitamins, and minerals). It was found that reduced DTAC may be considered an additional risk factor for developing type 2 diabetes mellitus (T2DM) [7].

The main sources of antioxidants in different populations are coffee, tea, fruits, and vegetables [8]. The health-promoting properties of fruits and vegetables are well known. However, coffee and tea are also sources of polyphenols and trace elements. Olechno et al. [9] showed that because coffee is widely consumed in many populations, it may be an additional source of manganese (up to 13.7% requirements per serving), zinc (up to 4.0% and 3.1% requirements per serving, respectively, for women and men), copper (up to 2.7% and 2.1% requirements per serving, respectively, for women and men) and iron (up to 0.4% and 0.6% requirements per serving, respectively, for women and men), which are components of antioxidant enzymes. Coffee infusions can also be a source of fluoride, chromium, and cobalt. The origin of coffee beans and the type of water used can affect the mineral content of the infusions. However, the brewing method does not play an important role. It is worth noting that the bioavailability of these coffee components is quite limited, especially in terms of iron.

The beneficial effect of polyphenols on the prevention and treatment of chronic diseases has been demonstrated in many studies. Polyphenols, whose source is mainly plant foods, are widely distributed in a human diet. An additional source of polyphenols are supplements.

Rodrigo-Gonzalo et al. [10] conducted a research review on the effect of polyphenols on cognitive function in the elderly. The authors have demonstrated that dietary supplementation with flavanols or stilbenes was associated with improvements in attention, psychomotor speed, delayed recall, and word recognition. However, the greatest benefits were found with higher doses and treatments of longer duration. Changes were generally assessed by cognitive tests or with magnetic resonance imaging. It was revealed that the type of cognitive test used to assess the effect of the intervention was critical. Several studies in this review also showed improvements in patients’ biochemical parameters and blood pressure.

Yang et al. [11], in an updated review, summarized the effects and mechanisms of curcumin on various cancers, based on the results of studies in cell and animal models as well as clinical trials. Curcumin is the main component of turmeric (Curcuma longa L.) and is widely used in the food industry. Curcumin has various biological activities, such as antibacterial, anti-inflammatory, antioxidant, and anticancer activities. Many studies have reported the effectiveness of curcumin in preventing and treating various cancers, such as thyroid, breast, gastric, colorectal, liver, pancreatic, prostate, and lung cancers. However, certain limiting factors, such as poor water solubility and very low oral bioavailability, may reduce its therapeutic effects. Several nanomaterials have been developed to prolong the release or targeted delivery of curcumin to tumor tissues, thereby enhancing the bioavailability and anticancer activity of curcumin. In addition, studies have shown that curcumin is generally safe and well tolerated, although some side effects, such as diarrhea and nausea, have been observed.

Alam et al. [12] presented evidence both in vitro and in vivo of the antioxidant role of apigenin, a natural flavonoid, in cardioprotection, neuroprotection, and renoprotection, as well as its beneficial effects on MetS and MetS-dependent organ dysfunction. Apigenin, as a bioactive compound derived from plant food (e.g., parsley, onion, oranges, chamomile, celery, spices, honey, wine, tea, and beer), can be used as a supplement or nutraceutical product; however, the isolation of bioactive compounds from food, their bioavailability and their stability pose difficulties. Therefore, apigenin must be evaluated in novel therapeutic formulations through nanodelivery and microdelivery techniques so as to enhance its therapeutic efficacy and target specificity.

Tresserra-Rimbau et al. [13] in the PREDIMED-Plus trial in Spain, assessed the relationship between changes in the intake of all polyphenol groups and T2DM-related parameters in a senior population with diabetes or at high risk of developing diabetes after one year of follow-up. This study involved 5921 participants aged 55–75 years. It was shown that the mean total polyphenol intake was 854 ± 318 mg/day at baseline and 855 ± 293 mg/day after one year, indicating no overall change. The most frequently consumed dietary polyphenols were flavonoids (58%) and phenolic acids (33%) in both stages of the study. Among the polyphenol class, the most consumed were hydroxycinnamic acids (30%), flavanols (27%), proanthocyanidins (24%), flavanones (10.6%), flavones (9%), flavonols (6%), anthocyanidins (5%), catechins (3%) and hydroxybenzoic acids (2%). The authors found no significant associations within the non-diabetic group, whereas in the prediabetic group, several polyphenol classes were inversely associated with levels of glucose (total polyphenols, total flavonoids, proanthocyanidins, flavanones, and flavones) or glycated hemoglobin HbA1c (flavones and lignans). Fewer polyphenol groups were associated with glucose-related parameters in diabetic participants (flavonols, lignans, and stilbenes). However, the authors noted that assessing the health benefits of polyphenol intake is complex due to their diverse chemical structure and variable bioavailability, the complexity of estimating their content in foods, their potential interactions with other nutrients, and the biological aspects that can modify their metabolism.

Choi et al. [14] studied the anti-allergic effect of the flavonol miquelianin (quercetin 3-O-glucuronide; MQL) in an ovalbumin (OVA)-induced Th2-dominant mouse model. MQL is an active compound in *Rosae multiflorae* fructus extract. Allergic diseases, including atopic dermatitis (AD), induce type 2 helper T (Th2) cell-dominant immune responses. Oral MQL suppressed cytokine and IL-2 production and proliferation of Th2 cells, and upregulated heme oxygenase-1 (HO-1) in splenocytes. Ex vivo, MQL suppressed Th1- and Th2-related immune responses by inhibiting CD4+ T cell proliferation, and upregulated HO-1 in CD4+ T cells by activating the C-Raf–ERK1/2–Nrf2 pathway via induction of reactive oxygen species’ generation. In a trimellitic anhydride-induced AD-like mouse model, both topical and oral MQL ameliorated AD symptoms by suppressing Th2 immune responses. These results suggest that MQL is a potential therapeutic agent for CD4+ T cell-mediated diseases, including allergic diseases.

Di Majo et al. [15] showed that in a male Wistar rat model, a metabolic syndrome (MetS) condition was induced following eight weeks of a high-fat diet (HFD) with a hepatic profile typical of nonalcoholic fatty liver disease (NAFD), altering glucose and lipid homeostasis and increasing visceral adipose tissue, but also impairing antioxidant/oxidant homeostasis. The authors assessed some biomarkers of oxidative stress, i.e., thiols balance, lipid peroxidation, and antioxidant barriers, and their correlations with clinical manifestations of MetS. Hepatic steatosis in association with the oxidative stress condition was also highlighted by histological analysis.

Lee et al. [16] investigated the potential role of hepatic miR-34a-5p and gallic acid (GA) in regulating hepatic lipid metabolism and diabetic steatosis in a mouse model. It was found that GA improved antioxidant enzyme activity and suppressed lipid accumulation in HFD-induced steatotic mouse liver. In vitro, the silencing of miR-34a-5p in hepatocyte HepG2 cells ameliorated the high glucose + oleic acid/palmitic acid mixture–induced accumulation of cellular triglycerides. The authors identified nuclear factor erythroid-derived 2-like 2 (NFE2L2) as a direct target of miR-34a-5p. Reduction of intracellular triglycerides and the expression levels of sterol regulatory element-binding protein 1 and fatty acid synthase by GA were mediated by the inhibition of miR-34a-5p expression in HepG2 cells. GA and an NFE2L2-activating agent could downregulate hepatic miR-34a-5p expression, and thus may be beneficial for treating diabetic steatohepatitis.

This Special Issue “Dietary Antioxidants and Chronic Diseases” published 11 papers, including 5 original research papers, 5 review articles, and 1 case report, all of which discuss various aspects of dietary antioxidants in chronic diseases. We thank all the authors for their high-quality contributions, *Antioxidants* for inviting us as guest editors, and all the reviewers for their diligent and responsible work. We hope that this Special Issue will add new content to the current knowledge on dietary antioxidants in various diseases and encourage further research.
